# Selection of patients with acute vertebrobasilar artery occlusion for endovascular treatment by magnetic resonance imaging

**DOI:** 10.3389/fneur.2023.1084819

**Published:** 2023-02-20

**Authors:** Jun Chen, Jiwei Zhang, Xianjin Zhu, Xuebin Zhang, Bin Jiang, Qi Liu, Zheng Z. Wei

**Affiliations:** ^1^Department of Geriatrics, The Affiliated Hospital of Chengde Medical University, Chengde, Hebei, China; ^2^Department of Neurosurgery, The Affiliated Hospital of Chengde Medical University, Chengde, Hebei, China; ^3^Department of Neurology, Beijing Friendship Hospital, Capital Medical University, Beijing, China; ^4^Department of Radiology, China-Japan Friendship Hospital, Beijing, China

**Keywords:** endovascular treatment, ischemic stroke, mechanical thrombectomy, diffusion-weighted imaging (DWI), acute vertebrobasilar artery occlusion

## Abstract

**Background and purpose:**

The best method for selecting patients with acute vertebrobasilar artery occlusion (VBAO) who would benefit from endovascular treatment (EVT) is still the key question. This study aimed to assess the efficacy of magnetic resonance imaging (MRI) for selecting patients with acute VBAO for EVT.

**Materials and methods:**

A total of 14 patients with suspected acute VBAO on MR angiography (MRA) in the EVT database (from April 2016 to August 2019) were enrolled. Acute Stroke Prognosis Early Computed Tomography Score (ASPECTS) and pons–midbrain index were assessed on diffusion-weighted imaging (DWI). EVT included a stent retriever and a rescue treatment (angioplasty and/or stenting). The proportion of successful reperfusion and favorable functional outcomes (modified Rankin Scale ≤ 3) at 90 days was documented.

**Results:**

A total of 11 patients were included in the final analysis. The median DWI-ASPECTS and pons–midbrain index were 7 and 2, respectively. Underlying stenosis was detected in 10 of 11 (90.9%) patients. Balloon angioplasty and/or stenting were used as rescue therapy for five patients and two patients, respectively. A total of nine patients (81.8%) achieved successful reperfusion (mTICI, 2b, or 3). The 90-day mRS score of 0–3 was achieved in six (54.5%) patients. The mortality rate within 90 days was 18.2% (two of 11 patients).

**Conclusion:**

DWI plus MRA could help select the patients with acute VBAO for EVT by assessing ASPECTS and the pons–midbrain index. Patients could achieve good reperfusion and favorable functional outcomes.

## Introduction

Basilar artery occlusion accounts for 5%−10% of all proximal intracranial occlusions and is associated with severe disability and death in up to 80% of patients, despite recent advances in the treatment of acute stroke (1). Recently, multiple randomized trials have shown the benefit of early recanalization with mechanical thrombectomy for treating acute ischemic stroke (AIS) due to large-vessel occlusion in the proximal anterior circulation (2). The result of endovascular treatment (EVT) for vertebrobasilar artery occlusion (VBAO) is still controversial.

Two preceding randomized trials, the Basilar Artery Occlusion Endovascular Intervention vs. Standard Medical Treatment (BEST) trial (3) and Basilar Artery International Cooperation Study (BASICS) trial (4), for comparing EVT vs. best medical management did not demonstrate significant differences in favorable functional outcomes at 90 days between the two groups. However, two recent randomized trials from China, namely, the Basilar Artery Occlusion Chinese Endovascular trial (BAOCHE) (5) and Endovascular Treatment for Acute Basilar Artery Occlusion(ATTENTION) (6), were reported with better functional outcomes and reduced mortality for EVT over best medical therapy alone. The best method for selecting patients with acute VBAO who would benefit from EVT is still the key question.

Imaging with the Acute Stroke Prognosis Early Computed Tomography Score (ASPECTS) was often used to identify patients who were most likely to be benefited from EVT in most trials (7, 8). In addition to ASPECTS, ischemic regions were also related to functional outcomes (9). Patients with acute VBAO could present with various symptoms from dizziness to coma, depending on the degree of involvement of the brainstem (10), even with similar ASPECTS. The pons–midbrain index was used to assess the degree of involvement of the brainstem in some studies (11, 12). Diffusion-weighted imaging (DWI) was regarded as the diagnostic “gold standard” for patients with posterior circulation (pc) stroke (12). Better imaging of the brainstem by DWI could help select a suitable patient for EVT. The combined use of the pc-ASPECTS and pons–midbrain index might help selection for the treatment of acute VBAO for EVT. We report a case series of AIS due to acute VBAO diagnosis with DWI and treated with EVT.

## Methods

### Patient selection

We reviewed our endovascular treatment (EVT) database (from April 2016 to August 2019) for patients with AIS. All patients signed informed consent before the operation. We included data of consecutive patients with AIS if they fulfilled the following criteria: (1) age 18–85 years; (2) symptoms suggest acute posterior ischemic stroke ≤ 24 h; (3) pre-stroke modified Rankin Scale (mRS) score ≤ 2; (4) baseline NIHSS ≥6; (5) an ability to provide informed consent. Patients were excluded from the study in the case of (1) being allergic to iodized contrast medium and unable to complete DSA examination; (2) without MR examination before EVT; (3) having acute cerebral hemorrhage; and (4) pregnancy.

This observational study was approved by the local institutional ethics committees. Demographics, clinical findings, imaging data, and risk factors such as hypertension, hyperlipidemia, diabetes mellitus, cigarette smoking, and atrial fibrillation were noted. After admission, the National Institutes of Health Stroke Scale (NIHSS) score of patients before thrombectomy was evaluated by stroke experts. The NIHSS score of patients with unconscious/coma was 38. The NIHSS score at admission and time information were documented.

### Imaging evaluation

Brain computer tomography (CT) scans were acquired for all patients upon hospital admission to exclude intracranial parenchymal hemorrhage or subarachnoid hemorrhage. The MRI modalities included two sequences: DWI and MRA. The ischemic core was assessed on DWI, and the pc-ASPECTS and the pons-midbrain index was calculated on DWI by two independent stroke experts. The site of VBAO was evaluated on MRA and confirmed on digital subtraction angiography (DSA). The degree of VBAO recanalization of patients submitted to endovascular treatment was classified using the modified Thrombolysis in Cerebral Infarction (mTICI) by two experienced neuroradiologists who were blinded to clinical outcome.

### Procedures

In this study, patients received intravenous alteplase within 4.5 h or intravenous urokinase within 6 h of stroke symptom onset. EVT was performed under either conscious sedation or general anesthesia according to the clinical condition of each patient.

A stent retriever was the preferred device for thrombectomy. Balloon angioplasty and/or stenting of the vertebral artery or basilar artery was allowed in the case of persistent occlusion or high-grade stenosis after thrombectomy. For patients without prior use of antiplatelet therapy, an antiplatelet loading dose (aspirin 300 mg + clopidogrel 300 mg) was given when the decision to proceed to stent placement was made. The successful reperfusion was defined as an mTICI 2b (50%−99% reperfusion) or 3 (complete reperfusion) at the end of all endovascular procedures. The maximum number of passes for devices was done according to the manufacturer's instructions.

### Follow-up and outcome

The primary outcome was the proportion of patients achieving successful post-procedure reperfusion (mTICI 2b-3), and favorable outcomes were defined as mRS score of 0–3 (with 0 meaning no symptoms, 1 able to do all usual activities, 2 able to look after own affairs without assistance but unable to do all previous activities, and 3 requires some help but able to walk unassisted) at 90 days. The secondary endpoints included functional independence (mRS score 0–2) at 90 days, and symptomatic intracranial hemorrhage (defined as evidence of intracranial hemorrhage on brain CT and an increase of 4 or more points on the NIHSS within 24 h).

### Statistical analysis

The intraclass correlation coefficient (ICC) with 95% confidence intervals (CIs) was used to assess the intraobserver or interobserver reliability for the evaluation of DWI-ASPECTS and pons–midbrain index. Cohen's k-statistic was computed to assess the observer reproducibility. Continuous variables were presented as mean ± standard deviation (SD) or median with interquartile range (IQR). Categorical variables such as male sex, risk factors, and degree of stenosis were presented as percentages. SPSS 11.5 (SPSS, Inc., Chicago, IL) was used as the statistical analysis software. All reported *p*-values were two-sided, and *p*-values < 0.05 were considered significant.

## Results

The data of 14 patients with suspected acute VBAO on MRA were reviewed. One patient with suspicious acute VBAO on MRA was finally diagnosed with severe stenosis of the basilar artery by DSA and excluded from this study. In total, 13 patients were finally confirmed as acute VBAO by DSA. Among these patients, two patients without management with EVT were also excluded in the final analysis due to unavailable arterial access to the bilateral vertebral arteries including one patient with chronic occlusion in the bilateral vertebral artery and another patient with chronic occlusion in the right vertebral artery and left vertebral artery hypoplasia. Eleven patients were included in the present analysis. The mean age was 56.9 ± 5.7 years, and eight (72.7%) were male patients. The most common baseline risk factors for stroke included hypertension (*n* = 10, 90.9%), diabetes mellitus (*n* = 6, 54.5%), cigarette smoking (*n* = 5, 45.5%), and atrial fibrillation (*n* = 0). The median initial NIHSS score was 20 (IQR: 8–38). One of 11 (9.1%) patients received intravenous thrombolysis with tPA.

**Figure 1:**
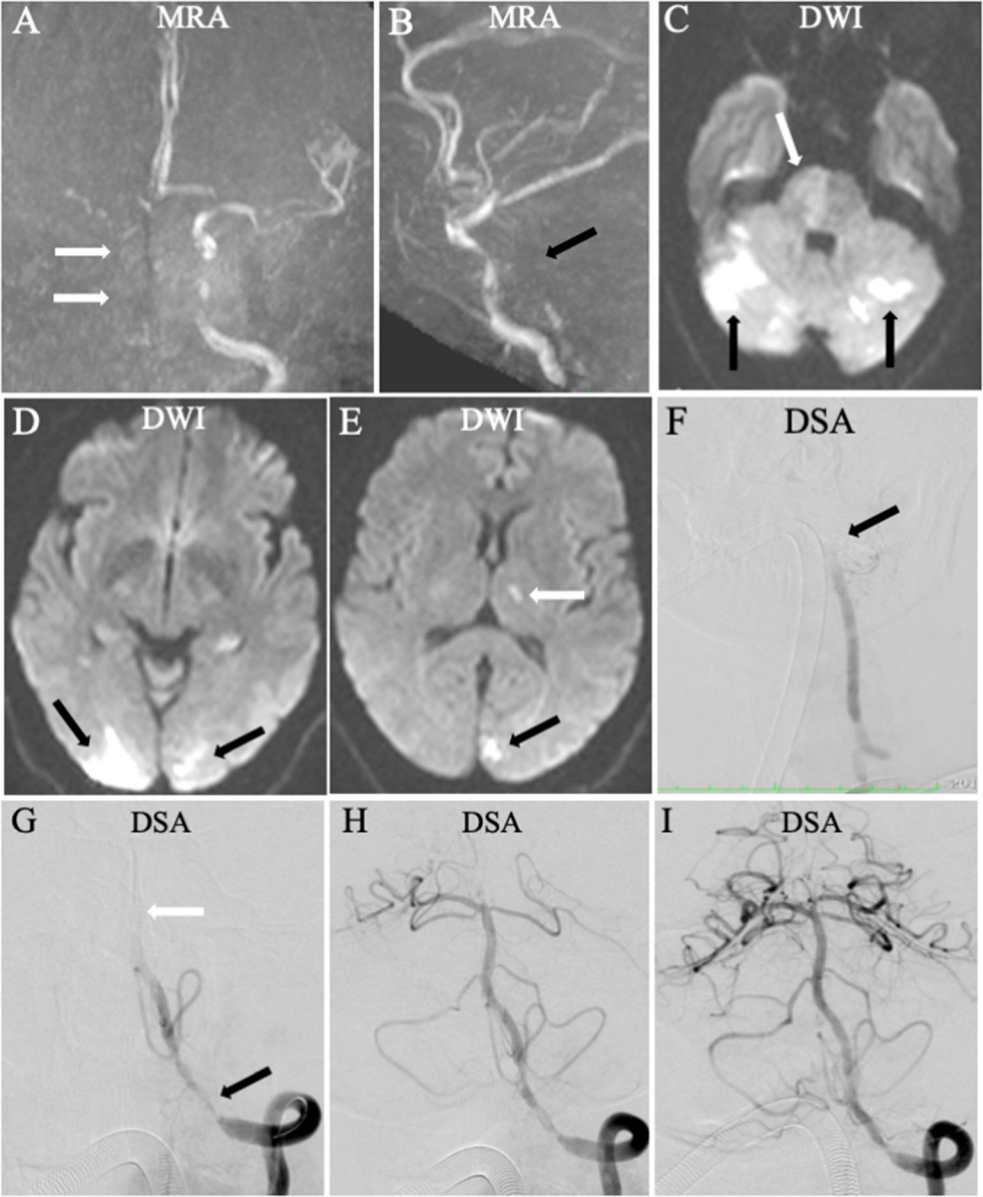
Endovascular treatment (EVT) for a patient with higher National Institutes of Health Stroke Scale (NIHSS) score: 20 and lower Acute Stroke Prognosis Early Computed Tomography Score (ASPECTS). Magnetic resonance angiography displayed the right internal carotid artery occlusion [**(A)**, arrow] and the vertebrobasilar artery occlusion [**(B)**, black arrow]. Diffusion-weighted imaging showed multiple acute ischemic stroke in posterior circulation involving the right pons [**(C)**, white arrow], bilateral cerebellum [**(C)**, black arrow], bilateral occipital lobe [**(D)**, black arrow], and left ganglion [**(E)**, white arrow]. The Acute Stroke Prognosis Early Computed Tomography Score (ASPECTS) and pons–midbrain index were 3 and 2, respectively. Based on the findings of DWI and MRA, the left vertebral and basilar artery was diagnosed as the “criminal” artery. Digital subtraction angiography (DSA) confirmed the artery occlusion for the left vertebral and basilar artery [**(F, G)**, white arrow). Endovascular treatment was performed with a stent retriever **(H)**, resulting in successful reperfusion with the modified thrombolysis in cerebral infarction 2b **(I)**. The modified Rankin Scale (mRS) score was 3 at 90 days.

The median DWI-ASPECTS was 7.0 (IQR, 6.0–7.0) with high intraobserver (ICC:0.977, 95% CI: 0.916–0.994) and interobserver agreement (ICC: 0.936, 95% CI: 0.763–0.983). An ischemic stroke involving the brainstem was found in nine patients with the median pons–midbrain index 2 (IQR: 2–3) with high intraobserver (ICC:0.966, 95% CI: 0.875–0.911) and interobserver agreement (ICC: 0.958, 95% CI: 0.843–0.989; Table 1, Figure 1).

**Table 1:** Procedural-associated characteristics of the patients.

**Patient no**.	**Occlusion site**	**Underlying site of stenosis**	**DWI-regions**	**DWI-aspects**	**Pons-midbrain-index**	**Pretreatment NIHSS**	**Onset-to- reperfusion time (min)**	**Treatment with IVT**	**Endovascular treatment**	**mTICI**	**mRS on 90-days**
1	BA	50%	L-pon, R-cerebellum	7	2	8	363	No	Solitaire	2b	0
2	BA	78%	B-pon, R-cerebellum	7	2	7	1074	No	Solitaire + angioplasty + apollo stent	3	0
3	BA	—	B-pon, L-midbrain	6	4	8		No	Solitaire	0	6
4	BA	80%	R-pon, R-cerebellum	7	3	12	328	No	Solitaire	2b	1
5	BA	80%	B-pon, R-occipital lobe	7	4	38	1226	No	2Solitaire + angioplasty	2b	4
6	Left V + BA	70%	B-pon, B-cerebellum	6	2	38	840	No	Solitaire + angioplasty	2a	6
7	BA	70%	L-pon, R-PCA territory	7	2	11	218	No	Solitaire	2b	3
8	Left V4 + BA	70%	R-pon, L-ganglion, B-cerebellum, B-PCA territory	3	2	20	509	No	Solitaire	2b	3
9	BA	75%	B-pon, B-cerebellum, L-PCA territory	5	3	20	804	No	Solitaire + angioplasty + stent	2b	5
10	BA	80%	L-ganglion, R-cerebellum, R-PCA territory	7	0	38	281	No	Solitaire	3	5
11	Right V4	50%	L-ganglion, L-cerebellum, L-PCA territory	7	0	24	466	Yes	Solitaire	3	2

BA, basilar artery; DWI, diffusion-weighted imaging; ASPECTS, Acute Stroke Prognosis Early Computed Tomography Score; NIHSS, National Institutes of Health Stroke Scale; mTICI, modified Thrombolysis in Cerebral Infarction; mRS, modified Rankin Scale.

Among the 11 patients, the median time from stroke onset to reperfusion was 487.5 min (IQR: 316.3–898.5 min). The median time from a puncture to successful recanalization was 36.5 min (IQR: 18.0–71.8 min). The median procedure time for patients with rescue therapy was 71.0 min (IQR: 55.5–86.5 min), which is longer than the median procedure time for patients without rescue therapy [19.0 min (IQR: 14.5–24.0) min, *p* = 0.009].

A stent retriever (Solitaire AB device) was used for all 11 patients. Underlying intracranial atherosclerosis (ICAS) was detected in 10 of 11 (90.9%) patients, including moderate stenosis in two patients, and severe stenosis in eight patients. Balloon angioplasty and/or stenting were then used as rescue therapy for four patients and two patients, respectively. Among the 11 patients treated with EVT, seven (63.6%) used a stent retriever (Solitaire AB device) alone, two (18.2%) were treated with EVT plus basilar or vertebral artery balloon angioplasty, and two (18.2%) were treated with EVT plus balloon angioplasty and stent (Table 1).

A total of ten patients (90.9%) achieved successful reperfusion (mTICI, 2b or 3). One patient did not achieve successful reperfusion (mTICI, 0) after multiple thrombectomy. The 90-day mRS score of 0–2 and 0–3 was achieved in four (36.4%) patients and six (54.5%) patients, respectively. The mortality rate within 90 days was 18.2% (two of 11 patients). Intracranial parenchymal hemorrhage and subarachnoid hemorrhage were found in one patient (9.1%) and two patients (18.2%), respectively.

## Discussion

This study showed that MRI with DWI and MRA could identify the occluded intracranial arteries and assess the ischemic size (ASPECTS) and regions (pons–midbrain index) for the patients with suspicious acute VBAO. Mechanical thrombectomy with a stent retriever plus rescue therapy (balloon angioplasty and/or stenting), when necessary, could quickly and effectively restore blood flow to the brain and improve the prognosis of patients with acute VBAO in the real world.

Previous studies showed ischemic core volume was independently associated with functional independence (13, 14). The optimal imaging methods for selecting patients who might benefit from EVT in acute ischemic stroke included CTP and DWI (15, 16). RAPID software could be used to assess the ischemic core in some large studies (7, 16) but might be unavailable in many hospitals due to its high price. ASPECTS was correlated with ischemic core volume and is often used for assessing the ischemic core in the real world (7, 15, 16). DWI was more sensitive than CT for detecting acute ischemic stroke (17). The pc-ASPECTS and pons–midbrain index could be evaluated on DWI with high intraobserver and interobserver agreement, which could help in the selection of suitable patients for EVT.

The stroke causative mechanism included ICAS, cardioembolism, and other or unknown reasons (17). ICAS-related occlusions were reported more frequently in the Asian population than in the Western population (3, 5, 6, 18), with unique risk factors. In the study, atherosclerotic risk factors were more frequently detected than atrial fibrillation (*n* = 0). The preoperative diagnosis of ICAS-related occlusion was still difficult with current imaging modalities. Residual stenosis of 50% or more after initial thrombectomy, or intraprocedural restenosis or reocclusion in the procedure was regarded as accepted diagnostic criteria of underlying ICAS-related occlusion (19, 20). Furthermore, the prevalence of ICAS-related vertebrobasilar occlusion was higher compared with anterior circulation, especially in Chinese patients (BEST, 52%; ANGEL-ACT Registry, 51%) (3, 18). In this study, underlying severe and moderate vertebrobasilar stenosis was found in eight (72.7%) and two (18.2%) of the 11 patients, respectively.

A stent retriever is recommended as the first-line device for mechanical thrombectomy. However, for the patients with IACS-related occlusion, good reperfusion might fail to be achieved by a stent retriever alone, possibly because of new thrombus formation induced by intimal injury from the passage of the stent retriever. The stent retriever could damage the plaque surface, resulting in increased platelet activation and thrombus formation, even reocclusion. Angioplasty and/or stenting have been considered as a rescue treatment for ICAS-related occlusions refractory to thrombectomy (18–21). In this study, four of eight patients with underlying severe stenosis could not achieve satisfactory reperfusion after initial stent retriever thrombectomy and received angioplasty and/or stenting.

In this study, the time from onset to reperfusion and the time from door to reperfusion were slightly longer than in the previous studies. The following reasons could help explain the findings. The procedure time would be longer for the patients with rescue therapy than for the patients without rescue therapy. A meta-analysis of studies of thrombectomy for ICAS-related occlusions showed atherosclerotic occlusions were associated with longer procedure times than non-atherosclerotic occlusion (20). In addition, diffusion MRI-selected patients for EVT were associated with a longer time than CTP-selected patients (15).

Our study has several limitations. First, this was a retrospective study with a small number of patients. A larger cohort study would be performed in the future. Second, there was no control group of patients for assessing the safety and efficacy of EVT.

## Conclusion

MRI with DWI and MRA could identify the occluded intracranial arteries and assess the ischemic size (ASPECTS) and regions (pons–midbrain index) for the patients with suspicious acute VBAO. Mechanical thrombectomy with a stent retriever, combined with rescue treatment of angioplasty and/or stenting, could achieve good reperfusion and favorable functional outcome for patients with acute VBAO.

## Data availability statement

The original contributions presented in the study are included in the article/supplementary material, further inquiries can be directed to the corresponding authors.

## Ethics statement

The studies involving human participants were reviewed and approved by the Ethics Committee of the China-Japan Friendship Hospital. Written informed consent for participation was not required for this study in accordance with the national legislation and the institutional requirements.

## Author contributions

JC, JZ, and XZhu: conceived and designed the research and made critical revisions of the manuscript. JC, JZ, XZhu, and XZha: acquired the data. JC, JZ, XZhu, XZha, BJ, and QL: analyzed and interpreted the data. JC, JZ, XZhu, and ZW: draft the manuscript. JC, JZ, XZhu, XZha, ZW, BJ, and QL: approved the final manuscript.
